# Recruiting Participants for Population Health Intervention Research: Effectiveness and Costs of Recruitment Methods for a Cohort Study

**DOI:** 10.2196/21142

**Published:** 2021-11-12

**Authors:** Rania Wasfi, Zoe Poirier Stephens, Meridith Sones, Karen Laberee, Caitlin Pugh, Daniel Fuller, Meghan Winters, Yan Kestens

**Affiliations:** 1 Centre for Surveillance and Applied Research Public Health Agency of Canada Ottawa, ON Canada; 2 Centre de recherche du CHUM Université de Montréal Montréal, QC Canada; 3 École de Santé Publique Université de Montréal Montreal, QC Canada; 4 Faculty of Health Sciences Simon Fraser University Burnaby, BC Canada; 5 School of Human Kinetics and Recreation Memorial University of Newfoundland St John’s, NL Canada

**Keywords:** recruitment methods, Facebook recruitment, cost-effectiveness, built environment, intervention research, natural experiment, mobile phone

## Abstract

**Background:**

Public health research studies often rely on population-based participation and draw on various recruitment methods to establish samples. Increasingly, researchers are turning to web-based recruitment tools. However, few studies detail traditional and web-based recruitment efforts in terms of costs and potential biases.

**Objective:**

This study aims to report on and evaluate the cost-effectiveness, time effectiveness, and sociodemographic representation of diverse recruitment methods used to enroll participants in 3 cities of the Interventions, Research, and Action in Cities Team (INTERACT) study, a cohort study conducted in Canadian cities.

**Methods:**

Over 2017 and 2018 in Vancouver, Saskatoon, and Montreal, the INTERACT study used the following recruitment methods: mailed letters, social media (including sponsored Facebook advertisements), news media, partner communications, snowball recruitment, in-person recruitment, and posters. Participation in the study involved answering web-based questionnaires (at minimum), activating a smartphone app to share sensor data, and wearing a device for mobility and physical activity monitoring. We describe sociodemographic characteristics by the recruitment method and analyze performance indicators, including cost, completion rate, and time effectiveness. Effectiveness included calculating cost per completer (ie, a participant who completed at least one questionnaire), the completion rate of a health questionnaire, and the delay between completion of eligibility and health questionnaires. Cost included producing materials (ie, printing costs), transmitting recruitment messages (ie, mailing list rental, postage, and sponsored Facebook posts charges), and staff time. In Montreal, the largest INTERACT sample, we modeled the number of daily recruits through generalized linear models accounting for the distributed lagged effects of recruitment campaigns.

**Results:**

Overall, 1791 participants were recruited from 3 cities and completed at least one questionnaire: 318 in Vancouver, 315 in Saskatoon, and 1158 in Montreal. In all cities, most participants chose to participate fully (questionnaires, apps, and devices). The costs associated with a completed participant varied across recruitment methods and by city. Facebook advertisements generated the most recruits (n=687), at a cost of CAD $15.04 (US $11.57; including staff time) per completer. Mailed letters were the costliest, at CAD $108.30 (US $83.3) per completer but served to reach older participants. All methods resulted in a gender imbalance, with women participating more, specifically with social media. Partner newsletters resulted in the participation of younger adults and were cost-efficient (CAD $5.16 [US $3.97] per completer). A generalized linear model for daily Montreal recruitment identified 2-day lag effects on most recruitment methods, except for the snowball campaign (4 days), letters (15 days), and reminder cards (5 days).

**Conclusions:**

This study presents comprehensive data on the costs, effectiveness, and bias of population recruitment in a cohort study in 3 Canadian cities. More comprehensive documentation and reporting of recruitment efforts across studies are needed to improve our capacity to conduct inclusive intervention research.

## Introduction

### Background

Urban interventions have the power to shape how people move, feel, and interact in cities, with the potential to improve health outcomes for all [1]. To understand the impacts of urban change on populations over time, researchers are using existing panel data sets [2-4] or collecting primary data [5,6]. Although representative population-based cohorts are key to successful population health intervention research [7], recruitment remains challenging [8,9]. Web-based recruitment strategies are increasingly used [10] because of their potential for wide reach over a short period and relatively low cost. Web-based technologies and related tools, such as smartphone apps or wearables, open new opportunities for data collection with lower participation burden. However, challenges to recruitment remain, including concerns over data privacy [11], time commitment for longitudinal studies [12], and limited reach toward marginalized populations [8,9]. All these can lead to biased samples, study delays, and increased costs [13].

Currently, few large-scale population health cohort studies have provided detailed reports on recruitment methods and effectiveness [6,14]. In a recent systematic review of studies that used Facebook to recruit participants in health, medical, or psychosocial research [10], only 19 out of 110 studies published between 2012 and 2017 reported details on cost and number of recruited participants by method. On average, the cost per completed participation through Facebook was CAD $6.79 (US $5.23; excluding staff time), although this varied widely (range CAD $1.36-$110 [US $1.05-$84.6]). Most of these studies were cross-sectional, with the exception of 2 cohort studies that focused on specific populations [15,16]. In a recent longitudinal web-based study examining physical activity through sustainable transport approaches in European cities, collaborations with local organizations, Facebook, mailing lists, and direct street recruitment were the most effective approaches to recruit participants, and Facebook was the most time-efficient method [6].

### Objective

The overarching aim of this paper is to report and evaluate the effect of different recruitment methods used to enroll participants in a cohort study in 3 Canadian cities, led by the Interventions, Research, and Action in Cities Team (INTERACT) [5].

## Methods

### Study Design and Procedures

INTERACT uses a longitudinal design that is currently applied to 4 Canadian cities: Montreal, Saskatoon, Vancouver, and Victoria [5]. Local teams aimed to recruit approximately 300-person samples, except for Montreal, where the initial goal was 3000 participants across the Montreal region, where we aimed to evaluate a larger set of built environment interventions.

In our analyses, we only concentrated on 3 of the 4 INTERACT cities: Montreal, Saskatoon, and Vancouver, where we asked participants to report on how they had heard about the study. Interested participants were invited to complete a web-based eligibility questionnaire after consenting to the study. Participants could identify how they had heard about the study, either through a letter in the mail, referral from a friend or family member, social media (eg, Facebook and Twitter), met with study team, website, or other. In Saskatoon, they could choose from a few additional specific options (eg, posters on buses). This information was used to run the analyses by the recruitment method.

Eligible participants could choose from different levels of participation. The participants were asked to complete two web-based questionnaires: a health questionnaire and the Visualization, Evaluation, and Recording of Itineraries and Activity Spaces (VERITAS), a map-based activity space and social network questionnaire [17,18]. In addition, participants could choose to download and activate a smartphone app collecting GPS and accelerometer data for 30 days and answer Ecologic Momentary Assessment of well-being for 7 days. They could also choose to wear a hip-worn multi-sensor device (SenseDoc; Mobysens Technologies) for 10 days.

### Target Sampling and Eligibility Criteria

Generally, participants were recruited through convenience sampling, with additional recruitment efforts aimed at reaching priority populations. Priority populations are those who are vulnerable or marginalized and need to be prioritized in research on healthy cities to ensure that every person has a fair and just opportunity to be as healthy as possible. Priority populations represent communities defined based on their age, gender, race, income, or ability. These priority populations include women, Black and Indigenous people, people with disabilities, people with low incomes, and older adults. For example, some social media campaigns specifically targeted underrepresented or low-income neighborhoods. The choice of inclusion criteria and survey questions was shaped by conversations with our knowledge user partners. Therefore, our recruitment approaches varied based on each site’s target sample and context. The specific recruitment tactics deployed in each city are described in the *Interventions and Participants by City* section. Inclusion criteria across all sites were as follows: being aged ≥18 years, being able to read or write English (or French in Montreal) well enough to answer a web-based questionnaire, and not planning to move out of the region in the next 2 years.

### Interventions and Participants by City

In Vancouver, INTERACT evaluates the impact of the Arbutus Greenway, a 9-km former railway being redeveloped into a continuous walking and cycling corridor. Recruitment was conducted from April 20 to September 20, 2018 (123 days). The initial inclusion criteria required participants to live in one of the 8 forward sortation areas (FSAs) within 2 km of Greenway and be aged ≥45 years. To boost recruitment and to be consistent with age limits used in other sites, recruitment was then extended from June 18 to 12 FSAs within 3 km of the Arbutus Greenway and to adults aged ≥18 years. Participants were entered into a lottery to win one of 5 CAD $50 (US $39.5) Visa gift cards and a CAD $600 (US $461.5) gift certificate for a stay at a resort hotel.

In Saskatoon, INTERACT is studying the impact of a Bus Rapid Transit (BRT) system. Inclusion criteria included riding the bus at least once in a typical month or living within 800 m of the proposed BRT lines as determined by their postal codes. Recruitment ran from September 19, 2018, to January 4, 2019 (108 days). The participants received a CAD $10 (US $7.69) gift certificate upon completion of the health questionnaire. To encourage participants to contribute more data, participants were entered into a prize draw and received an additional chance of winning for each additional level of participation (VERITAS Questionnaire, app, or SenseDoc). Prizes included transit passes, a Bluetooth speaker, and headphones.

In Montreal, INTERACT evaluated the impacts of built environment interventions related to the Montreal Sustainability Plan (Plan Montréal durable 2016-2020). Interventions of interest include traffic calming measures, new transportation infrastructure, place-making, and greening programs. Target areas for recruitment included the Island of Montreal, Longueuil, Brossard, Saint-Lambert, and Laval. Participants were recruited between June 6 and December 21, 2018 (199 days). Participants were entered into a prize draw, with 20 CAD $100 (US $76.9) Visa gift cards and 1 prize with a value of CAD $500 (US $384.6): a choice of an iPad, a bicycle, or a stay at a resort hotel. Similar to Saskatoon, participants’ chances of winning increased with their level of participation.

### Recruitment Methods

Recruitment methods deployed at all sites included social media, news media, partner communications, snowball recruitment, and other methods, including in-person recruitment activities. Specific efforts and opportunities were tailored to each city.

#### Mailed Letters

Mailed letters were sent to Vancouver and Montreal. Mailing lists were rented from Canada Post. For the initial recruitment in Vancouver, 8614 personalized letters with an accompanying bookmark were sent to all homes in the 8 FSAs within 2 km of the Greenway where an individual aged ≥45 years lived. In Montreal, a mailed letter campaign with 3 types of options was sent to 15,000 people: a personalized letter with a postcard followed by a reminder postcard 2 weeks later (n=5000; group A), a personalized letter with a color card without a reminder (n=5000; group B), or a nonpersonalized postcard only (n=5000; group C). Letters were sent out by a third-party mail provider from the Canada Post Marketing program. Mailings were stratified by postal code to enable group identification based on the participants’ postal code.

#### Social Media

All 3 cities used the INTERACT Twitter account (@teaminteract) and Facebook page [19] for promotion. In Montreal, the Centre de recherche du CHUM Facebook account also posted INTERACT content. In an effort to recruit underrepresented groups, messaging was adapted to younger people, and Facebook advertising was boosted in low-income postal codes in Montreal and Saskatoon. Facebook group administrators of community groups and nonprofit organizations in Montreal were contacted to post an invitation to the study.

#### News Media

Across all sites, the study was advertised through unpaid media coverage, through press releases to local media outlets, and by contact with journalists. In Montreal, the study was featured on news outlets such as *La Presse* and *Le Devoir*, CBC Montreal, *Montreal Gazette*, and TVA Nouvelles. In Saskatoon, the study was featured on CTV local news and CBC Saskatoon. In Vancouver, local CBC radio shows covered the study.

#### Newspaper Advertisement

In Montreal, information about the study was published in the Société de transport de Montréal section of the *Journal Métro*, free of charge.

#### Partner Communications

The research staff leveraged partner mailing lists, newsletters, and web-based spaces to promote the study. Efforts were made to reach community organizations working closely with citizens. Local teams also took advantage of institutional networks to share information, such as using listservs and university portals to advertise the study.

#### Snowball Recruitment

In Vancouver and Montreal, *Refer a friend* campaigns were launched using MailChimp. The participants were sent an email to share with a friend. Participants received a CAD $10 (US $7.69) gift card for every 2 referred friends who had signed up. In Saskatoon, participants were encouraged to share information about the study in their network, although no incentive was provided.

#### Other

We participated in a variety of community events to promote this study. In Saskatoon, research staff distributed flyers at bus terminals. In Vancouver, research staff attended farmers’ markets, street parties, and seniors’ activities around Arbutus Greenway. In Montreal, the team participated in city and community events, distributed flyers at the Centre hospitalier de l’Université de Montréal, and visited local food banks. At these events, we collected email addresses for follow-up with interested people. All 3 cities designed and distributed posters to advertise the study. In Vancouver, posters were placed in cafés, local shops, and community spaces. In Saskatoon, posters were placed on buses. In Montreal, posters were placed in universities, community centers, and municipal buildings.

### Recruitment Effectiveness Metrics

#### Cost

To calculate the cost of each recruitment method, we added the cost of producing materials (ie, printing costs), transmitting recruitment messages (ie, mailing list rental, postage, and sponsored Facebook post charges), and staff time. Staff time was assessed as 0.5 hours per Facebook post, 4 hours per in-person event, 2 hours per media publication, 2 hours per partner post, 50 hours for the mailed letters, and 35 hours for the snowball campaign. Compensation and expenses for prizes were excluded from the cost, as they were not consistent across sites.

#### Sociodemographic Profiles

We provide descriptive statistics on recruited populations for each method for age (4 categories: 18-34 years, 35-54 years, 55-64 years, and 65-88 years); gender (man, woman, and other); household income (CAD $0-49,999 [US $0-38,460]; CAD $50,000-99,999 [US $38,461-$76,922]; and CAD $100,000 [US $76,923] or more); education (less than a university degree, university degree, and graduate degree); and ethnicity (White; Indigenous or Aboriginal; and visible minorities, including South Asian, Chinese, Black, Filipino, Latin American, Arab, Southeast Asian, West Asian, Korean, and Japanese).

#### Effectiveness

Recruitment method–specific effectiveness was determined by calculating the cost per completer, completion rate of the health questionnaire, and completion delay. The completion rate was calculated as the number of people who completed the health questionnaire divided by the number of eligible participants. Completion delay is defined as the time between the completion of eligibility and the health questionnaires.

### Statistical Analyses

City differences in completion rate and completion delay were tested using the Kruskal–Wallis rank-based nonparametric method. A pairwise Wilcoxon test was used for multiple pairwise comparisons. Cost and compliance analyses per recruitment method were calculated for each city.

Modeling of daily recruitment by method and intensity was conducted for Montreal, where recruitment activities were recorded daily, and the sample size was larger. We modeled the number of participants recruited each day from the start to the end of the recruitment period (n=199 days). A recruited person was defined as someone who had completed the eligibility questionnaire and was deemed eligible and accepted to participate. Recruited participants were chosen over those who had completed the health surveys (eg, *completers*, above) to identify how different recruitment methods were able to reach participants and obtain their willingness to participate.

We fitted a distributed lag model using generalized linear regression to estimate the number of participants recruited on any given day. Predictive variables for each day were the type and intensity of recruitment campaigns, which included the following: (1) mailed letters, (2) people reached through paid Facebook posts and advertisements, (3) unpaid Facebook posts, (4) mailed reminders, (5) partner communications, (6) snowball recruitment campaigns, (7) wide-reach news media coverage (articles published in *La Presse* and *Le Devoir*, the 2 major francophone newspapers in Montreal), (8) smaller-reach news media coverage, and (9) other means of recruitment, including person events, posters, advertisements on web-based venues such as university websites, and classified advertisements. To consider the potential lag effect in recruitment for each method, we built different finite distributed lag weights ranging from 1 to 15 days [20]. Semilog transformations of the distributed lagged variables were used for Facebook reach:

y_d_ = α + β_1_ × [x1(d-s)] + β_2_ × [log x2(d-s)] + β_3_ × [x3(d-s)] + β_4_ [x4(d-s)] + β_5_ [x5(d-s)] + β_6_ [x6(d-s)] + β_7_ [x7(d-s)] + β_8_ [x8(d-s)] + β_9_ [x9(d-s)] +µ_t_

where y_d_=number of participants recruited on a given day, lag length (s)=1, 2, 3....q, *x*1=mailed letters, *x*2=paid Facebook reach/1000, *x*3=unpaid Facebook posts, *x*4=mailed reminders, *x*5=partner communications, *x*6=snowball recruitment campaigns, *x*7=wide-reach news media coverage, *x*8=smaller-reach news media coverage, and *x*9=other means.

We retained the most efficient lag length (number of days) for each campaign type based on statistical significance, model fit (Akaike Information Criteria and Bayesian Information Criteria), and R-squared. We also visually examined the distribution of the residuals by plotting the observed and predicted estimates. Full details on the construction of the lagged variables and results of all combinations of different lag lengths (summarized in a CSV file) are provided on INTERACT’s GitHub account [21]. Multimedia Appendix 1, Table S1 provides an example of 2- and 4-day lagged intensity variables. RStudio (version 3.6.1) was used to conduct all statistical analyses.

## Results

**Figure 1:**
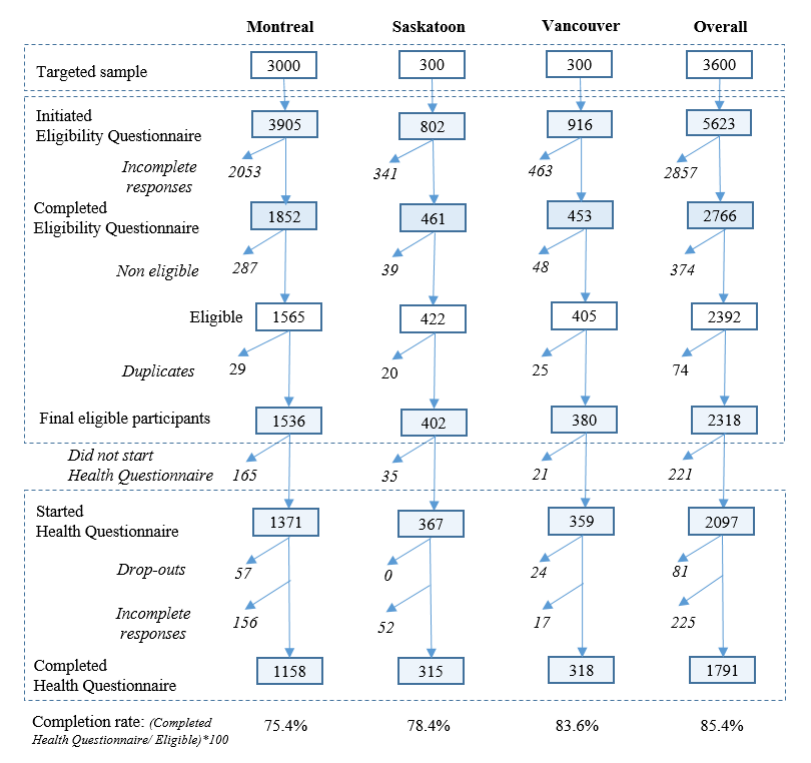
Flowchart of recruitment numbers in the 3 Interventions, Research, and Action in Cities Team (INTERACT) cities.

The recruitment flowchart (Figure 1) provides details of recruitment, dropouts, eligibility, and completion of the health questionnaire. Participation choices by city are presented in Table 1.

**Table 1:** Overall recruitment: participation option and health questionnaire completion by city^a^.

			Montreal	Saskatoon	Vancouver
Total number of recruited participants per city, N (%)			1536 (100)	402 (100)	380 (100)
Total number of recruited participants who completed the health questionnaire per city, n/N (%)			1158/1536 (75.4)	315/402 (78.4)	318/380 (83.7)
**Participation option**					
	**1. Full participation (with a smartphone app and multi-sensor device)**				
		Number of recruited participants, N_1_/N (%)	937/1536 (61)	225/402 (56)	161/380 (42.4)
		Participants who completed a health questionnaire, n/N_1_ (%)	744/937 (79.4)	179/225 (79.6)	134/161 (83.2)
	**2. Intermediate participation (with a smartphone app)**				
		Number of recruited participants, N_2_/N (%)	277/1536 (18)	67/402 (16.7)	68/380 (17.9)
		Participants who completed health questionnaire, n/N_2_ (%)	201/277 (72.6)	56/67 (84)	51/68 (75)
	**3. Intermediate participation (with multi-sensor device)**				
		Number of recruited participants, N_3_/N (%)	N/A^b^	13/402 (3.2)	72/380 (18.9)
		Participants who completed health questionnaire, n/N_3_ (%)	N/A	9/13 (69)	68/72 (94)
	**4. Basic participation (only questionnaires)**				
		Number of recruited participants, N_4_/N (%)	322/1536 (20.9)	97/402 (24.1)	79/380 (20.8)
		Participants who completed health questionnaire, n/N_4_ (%)	213/322 (66.1)	71/97 (73.2)	65/79 (82.3)

^a^The percentage of participants who completed the health questionnaire is provided per participation option.

^b^N/A: not applicable.

### Recruitment Methods and Corresponding Sociodemographic Profiles

Table 2 provides sociodemographic information using the recruitment method. City-specific numbers are provided in Multimedia Appendices 2 and 3. Most participants were recruited through social media (n=687). The participants were younger (mean age 41.8 years, SD 14.1, years) than those recruited through most other means, especially traditional media (mean age 58.5 years, SD 12.6, years). Other methods were more effective in recruiting younger participants, such as partner communications (mean 35.2 years, SD 16.1, years) or snowball sampling (mean 39.5 years, SD 15.1, years), compared with social media. Gender imbalance was strong across all methods, with 69% of all recruits identifying as women, 30% as men, and less than 1% as other genders. Social media recruitment was the most gendered (78% women vs 21% men vs 0.4% other) and letters the least (57% women vs 43% men). Recruits were distributed across income categories, with the highest share of lower-income participants (less than CAD $50,000 per year [US $38,461 per year]) recruited through partner communications (40%), *other* methods (32%), and social media (29%). All methods managed to recruit higher-income brackets (15.2% of the sample had household incomes equal to or above CAD $150,000 per year (US $115,384 per year), but this was particularly strong for mailed letters (22% of recruits by that category). Finally, most of the people in the sample identified as White (83.6%), followed by visible minorities (South Asian, Chinese, Black, Filipino, Latin American, Arab, Southeast Asian, West Asian, Korean, and Japanese: 13.6%), and Indigenous (1.6%). There were differences in proportions among the cities: in Montreal and Vancouver, only 10.1% and 15.1% were visible minorities and 0.4% and 1.3% were Indigenous, whereas Saskatoon’s sample consisted of 24.8% of visible minorities and 6.3% of Indigenous participants. Interestingly, in Saskatoon, 38.9% of the visible minority participants were recruited through partner communication.

**Table 2:** Demographic characteristics by recruitment method^a^.

Demographics		Mailed letters (n=282)	Social media (n=687)	News media (n=230)	Partner communications (n=218)	Snowball recruitment (n=121)	Other (n=253)	Total (n=1791)
**Age category (years), n (%)**								
	18-34	10 (3.5)	238 (34.6)	44 (19.1)	108 (49.5)	58 (47.9)	56 (22.1)	514 (28.7)
	35-54	91 (32.3)	273 (39.7)	74 (32.2)	47 (21.6)	34 (28.1)	75 (29.6)	594 (33.2)
	55-64	89 (31.6)	95 (13.8)	62 (27)	17 (7.8)	11 (9.1)	29 (11.5)	303 (16.9)
	65-88	92 (32.6)	48 (7)	46 (20)	13 (6)	12 (9.9)	39 (15.4)	250 (13.9)
**Education, n (%)**								
	Less than university degree	94 (33.3)	142 (20.7)	26 (11.3)	63 (28.9)	21 (17.4)	74 (29.2)	420 (23. 5)
	University degree	85 (30.1)	259 (37.7)	90 (39.1)	79 (36.2)	51 (42.1)	82 (32.4)	646 (36.1)
	Graduate degree	100 (35.5)	282 (41)	113 (49.1)	70 (32.1)	49 (40.5)	94 (37.2)	708 (39.5)
**Gender, n (%)**								
	Male	120 (42.6)	144 (21)	84 (36.5)	67 (30.7)	45 (37.2)	81 (32)	541 (30.2)
	Female	161 (57.1)	537 (78.2)	144 (62.6)	149 (68.3)	75 (62)	170 (67.2)	1236 (69)
	Other	0 (0)	3 (0.4)	2 (0.9)	2 (0.9)	0 (0)	2 (0.8)	9 (0.5)
**Income category, n (%)**								
	CAD $0- $49,999 (US $0- $38,460)	50 (17.7)	200 (29.1)	36 (15.7)	87 (39.9)	31 (25.6)	82 (32.4)	486 (27.1)
	CAD $50,000- $99,999 (US $38,461-$76,922)	79 (28.0)	212 (30.9)	91 (39.6)	38 (17.4)	41 (33.9)	56 (22.1)	517 (28.9)
	CAD $100,000-$149,999 (US $76,923-$115,383)	49 (17.4)	122 (17.8)	49 (21.3)	33 (15.1)	20 (16.5)	45 (17.8)	318 (17.8)
	CAD $150,000-$199,999 (US $115,384-$153,845)	29 (10.3)	53 (7.7)	19 (8.3)	14 (6.4)	9 (7.4)	22 (8.7)	146 (8.2)
	≥CAD $200,000 (US $153,846)	33 (11.7)	40 (5.8)	15 (6.5)	15 (6.9)	10 (8.3)	13 (5.1)	126 (7)
**Ethnicity, n (%)**								
	White	246 (87.2)	591 (86.0)	217 (94.3)	155 (71.1)	93 (76.9)	195 (77.1)	1497 (83.6)
	Indigenous or Aboriginal	<5 (0.7)	10 (1.5)	0	5 (2.3)	<5 (0.8)	11 (4.3)	29 (1.6)
	Visible minorities	31 (11)	81 (11.8)	11 (4.8)	55 (25.2)	23 (19)	42 (16.6)	243 (13.6)

^a^Missing responses: age: 7.25% (130/1791); education: 0.95% (17/1791); gender: 0.28% (5/1791); income: 11.05% (198/1791); and ethnicity: 1.23% (22/1791).

### Questionnaire Completion

Completion rate, calculated as the proportion of eligible recruits who completed the health questionnaire, varied by city and by recruitment method (Table 3). The completion rate was highest for Vancouver (83.6%) and lowest for Montreal (75.4%; Figure 1). The completion rate by recruitment method varied from 88.4% (mailed letters) to 72.5% (snowball recruitment), yet between-city variations were also observed. For example, Vancouver’s completion rate for those recruited through letters was 97.1%, compared with 87.1% in Montreal. The time elapsed between eligibility and health questionnaire completion varied widely across participants and recruitment methods but did not differ between cities. Those recruited through letters were quickest to complete the questionnaires (mean 9.1 days, SD 29.9, days), whereas the slowest were those recruited through social media (mean 14.3 days, SD 37.7, days), followed by partner communications (mean 13.8 days, SD 31.4, days), media (mean 11.8 days, SD 34.6, days), and other means (mean 10.6 days, SD 25.8, days).

**Table 3:** Completion of eligibility and health questionnaires and time taken by recruitment method for baseline INTERACT^a^ in Montreal, Saskatoon, and Vancouver.

	Recruitment method						
	Mailed letters	Social media	News media	Partner communications	Snowball recruitment	Other	Total
Number of participants who completed eligibility questionnaire (recruited), n (%)	319 (13.76)	944 (40.73)	284 (12.25)	264 (11.39)	167 (7.20)	340 (14.67)	2318 (100)
Number of participants who completed health questionnaire (completer), n (%)	282 (15.74)	687 (38.36)	230 (12.84)	218 (12.17)	121 (6.75)	253 (14.13)	1791 (100)
Average days from eligibility to completion of health questionnaire, mean (SD)	9.1 (29.9)	14.3 (37.7)	11.8 (34.6)	13.8 (31.4)	9.6 (28.6)	10.6 (25.8)	12.3 (10.4)
Completion rate, %	88.4	72.8	81.0	82.6	72.5	74.4	77.3

^a^INTERACT: Interventions, Research, and Action in Cities Team.

### Cost-effectiveness

Cost per completer by recruitment method varied by city (Table 4). The average cost per completer for the 3 cities (Montreal, Saskatoon, and Vancouver) was CAD $23.28 (US $17.91). City-specific costs per completion were CAD $26.52 (US $20.4) in Montreal, CAD $23.80 (US $18.3) in Vancouver, and CAD $10.85 (US $8.35) in Saskatoon. Cost per completer by recruitment method varied by city. Partner communications was the most cost-effective recruitment method across cities, with an average cost of CAD $5.16 (US $3.97) per completer. They were particularly efficient in Saskatoon, costing <CAD $1(US $1.3) per completer. News media cost on average CAD $7.35 (US $5.65) per completer and generated a considerable number of participants in Montreal.

Social media, which generated the most recruits, came third in terms of cost-effectiveness across cities, at an average cost of CAD $15.04 (US $11.57) per completer. The highest recruitment cost resulted from mailed letters, at an average of CAD $108.30 (US $83.3) per completer (CAD $130.80 (US $100.6) in Montreal; CAD $83.56 (US $ 64.27) in Vancouver). Comparing different mailed options showed that personalized letters were much more cost-effective than postcards only, and reminder cards did not help recruitment. The cost per completer for group B (personalized letter and color card only; n=88) was CAD $60.11 (US $46.23), followed by group A (personalized letter, color card, and a reminder postcard; n=75) at CAD $106.68 (US $82.06), and group C (nonpersonalized postcard only) was the costliest at CAD $796.34 (US $612.57) per completer (n=8 recruitment).

**Table 4:** Cost per completer by city and recruitment method.

Reported recruitment method			Montreal (n=1158)	Saskatoon (n=315)		Vancouver (n=318)		Total (n=1791)
**Mailed letters, n (%)^a^**			148 (12.8)	0 (0)		134 (42.1)		282 (15.7)
	Cost per completer, CAD$ (US$)^b^	130.80 (100.61)		N/A	83.56 (64.27)		108.30 (83.31)	
**Social media, n (%)**			503 (43.4)	88 (27.9)		96 (30.2)		687 (38.4)
	Cost per completer, CAD$ (US$)	13.35 (10.27)		16.13 (12.4)	22.91 (17.62)		15.04 (11.56)	
**News media, n (%)**			226 (19.5)	4 (1.3)		0 (US 0)		230 (12.8)
	Cost per completer, CAD$ (US$)	6.17 (4.75)		74.04 (56.95)	N/A		7.35 (5.65)	
**Other, n (%)**			109 (9.4)	79 (25.1)		65 (20.4)		253 (14.1)
	Cost per completer, CAD$ (US$)	18.05 (13.88)		21.60 (16.62)	72.70 (55.92)		33.20 (25.54)	
**Partner communications, n (%)**			91 (7.9)	126 (40)		1 (0.3)		218 (12.2)
	Cost per completer, CAD$ (US$)	7.32 (5.63)		0.88 (0.68)	347.10 (267)		5.16 (3.97)	
**Snowball recruitment, n (%)**			81(7)	18 (5.7)		22 (6.9)		121 (6.8)
	Cost per completer, CAD$ (US$)	16.40 (12.6)		0 (0)	59.80 (46)		21.85 (16.8)	
Average cost per completer, CAD$ (US$)			26.52 (20.4)	10.85 (8.35)		23.80 (18.3)		23.28 (17.9)

^a^Number of participants who completed the health questionnaire Percentages indicate the proportion of city participants recruited through this specific method.

^b^Cost per completer includes the cost of all materials, expenses, and staff time and is expressed in Canadian dollars. Additional costs of participant compensation in Saskatoon (CAD $10 [US $7.69]) for questionnaire completion) are not included in this table.

### Recruitment Modeling in Montreal

We modeled the number of people recruited per day over the 199-day recruitment period, which included 1536 participants from the Montreal cohort who completed the eligibility questionnaire and were willing to participate. The predictor variables included campaign events by recruitment type. Within the 199-day recruitment period, there were 227 campaign events, including (1) 151 days of paid Facebook posts and advertisements with an average reach of 2770 (SD 3558) potential participants (minimum=144, maximum=20,156); (2) 44 unpaid Facebook posts posted over 34 days; (3) a mailed letter campaign reaching 15,000 people; (4) a mailed reminder campaign reaching 5000 people; (5) 18 communications with partners who sent out newsletters or shared information on their web-based spaces; (6) 2 wide-reach media coverage events; (7) 6 smaller-reach media coverage events; (8) 1 snowball recruitment campaign; and (9) 16 other events, including 2 in-person community events.

**Figure 2:**
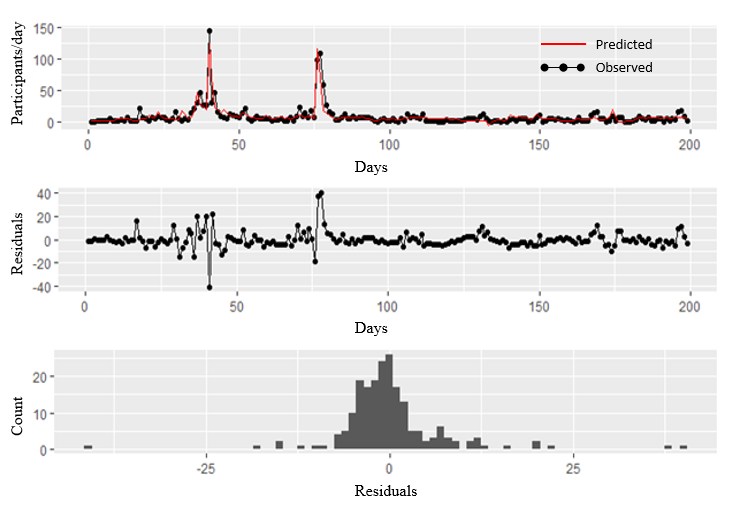
Predicted and observed daily recruitment of Montreal’s Interventions, Research, and Action in Cities Team (INTERACT) cohort.

The model performed relatively well overall, with an adjusted R-square of 0.78. The model parameters are listed in Table 5. Each coefficient should be interpreted as the effect of a campaign event or the number of participants recruited. The weights for each campaign are distributed over several days (specific lag per campaign type), and the sum of weights per campaign event equals 1. The model estimates 107 recruits that occur over 15 days for the letter campaign sent to 15,000 participants. Every 1% increase in Facebook reach per 1000 participants resulted in a 0.014 increase in recruitment. For example, an increase of 10% (277 participants) from the average Facebook reach of 2770 participants per day recruited an estimated 3.8 participants over 2 days. Unpaid Facebook posts recruited an estimated 3.2 participants over 2 days. On average, every wide-reach news article was associated with the recruitment of 164 participants over the course of 2 days. Finally, on average, each smaller-reach news media campaign was associated with an estimated recruitment of 10 participants. The best model fit and residual distribution indicated 2-day lag effects for all recruitment methods, except for 4 days for snowball recruitment, 15 days for the mailed letter campaign, and 5 days for the mailed reminder campaigns. Figure 2 shows the predicted and observed daily recruitment during the 199-day recruitment period.

**Table 5:** Results of the regression model estimating the number of participants recruited per campaign event (number of observations=199)^a^.

Predictors (lag in days)	Estimated number of recruited participants per campaign event	*P* value	95% CI
Mailed letters (15 days)	106.5^b^	.002	39.6 to 173.4
Mailed reminders (5 days)	16.9	.28	−13.5 to 47.2
Log paid Facebook reach per 1000 (2 days)	1.4^b^	<.001	0.6 to 2.1
Facebook unpaid posts (>2 days)	3.2^b^	.002	1.5 to 5.0
Wide-reach news media coverage (>2 days)	163.6^b^	<.001	149.0 to 178.2
Other recruitment means (>4 days)	−1.3	.05	−2.7 to 0.0
Partner communications (>2 days)	3.5	.16	−1.4 to 8.4
Snowball recruitment (>4 days)	2.5	.85	−24.4 to 29.3
Smaller-reach news media coverage (>2 days)	10.4^b^	.001	4.1 to 16.8
Intercept	4.3^b^	<.001	2.9 to 5.7

^a^R^2^/R^2^ adjusted=0.79/0.78.

^b^*P*<.05.

## Discussion

### Study Significance

This study documents the procedures and effectiveness of recruitment efforts to constitute baseline population-based samples in 3 Canadian cities as part of the INTERACT study. We further propose a daily recruitment modeling strategy that provides estimates of effectiveness for various recruitment campaigns, which we applied to the 199-day Montreal recruitment period. The existing literature generally lacks detailed reporting on recruitment performance [8,22]. To our knowledge, this is the first Canadian study to provide detailed performance indicators, including the time- and cost-effectiveness of different population-based recruitment methods.

### Recruitment Method and Cost-effectiveness

Social media is a powerful and relatively cost-effective way to recruit participants. Approximately one-third of our participants were recruited through social media (687/1791, 38.4%), at an average cost of CAD $15.04 (US $11.56) per participant. However, these participants also took the longest to complete the questionnaires and had the lowest completion rates. This is possibly because social media users have direct access to the web-based recruitment material and therefore are more inclined to start the process, even with medium levels of motivation.

In contrast, letters had the highest and fastest completion rates (Table 6). Even if few of those receiving a letter were engaged, those who did were committed. Researchers recruiting samples for longitudinal studies or for studies requiring substantial time commitments from participants may want to consider such trade-offs and plan for potentially different follow-up rates by recruitment strategy. Previous studies found that follow-up rates were generally lower when participants were recruited on the web [23,24]. Although this study reports only baseline recruitment, future work should also consider differential attrition rates linked to the different recruiting methods.

The effectiveness of social media for recruitment has increased over the years. Montreal’s (CAD $10.18 [US $7.83]) and Saskatoon’s (CAD $14.45 [US $11.11]) social media costs (excluding staff time to facilitate comparisons with the literature) are in line with previously reported median costs of CAD $11.60 (US $8.92) for Facebook recruitment across 18 studies [10]. In Vancouver, where we initially targeted older adults living in a small geographic area, social media costs were higher (CAD $21.30 [US $16.38]) but in line with a recent Canadian study that recruited a hard-to-reach population through Facebook (CAD $19.27 [US $14.82]) [25].

Facebook posts were reported as an efficient recruitment method for a cohort study across 7 European cities (recruitment period: 2014-2016), although the cost per completer was not documented [6]. Earlier reports on one of the largest prospective cohort studies in the United Kingdom (recruitment period: 2009-2012) showed that Facebook posts are less efficient than mailed letters, SMS text messages sent on mobile phones, and emails [12]. However, since 2009, the share of the population with a social media account has grown, and Facebook has considerably refined its advertisement program, facilitating reach and recruitment [26]. Facebook advertisement features now make it possible to specifically target local areas or population segments based on individual profiles. These tools allow research teams to react to potential biases during the recruitment process, for example, by adjusting campaigns by targeting underrepresented geographic areas or population groups. Concomitantly, physical mail use has diminished, at least for letter correspondence. A previous study on smoking targeting young adults in Montreal (recruitment: 2011-2012) reported a 25% participation rate through letter recruitment [27], a much higher number than was achieved here (<1%). This difference might be linked to the presence of compensation and the age of the participants because CAD $10 (US $7.69) gift certificates were given to those completing the survey. Transformations in communication habits and lower receptivity for mailed communication may also partly explain this difference. Finally, letters recruited older men than other methods, whereas social media recruited younger women, meaning these methods may be complementary.

Garnering attention for the study through newspaper articles was the second most effective strategy in Montreal, recruiting a high share of participants (226/1158, 19.5%) at a low cost (CAD $6.17 [US $4.75] per participant including staff time). Opportunities to publicize public health research in mainstream news outlets should be seized not only as a way to reach future participants, but also as a means to highlight existing research on the topic.

We recommend that researchers use multiple recruitment methods to amplify the impact of messaging and reach a greater diversity of participants. In Montreal, social media recruits were younger (mean age 41.8 years, SD 14.1, years) than those recruited through letters (mean age 58.8 years, SD 12.6, years) and media campaigns (mean age 51.4 years, SD 14.8, years). However, our social media recruitment profiles echo Canada’s Facebook users: 38.2% (192/503) of our social media recruits were aged 18-34 years (42% of Facebook users in Canada), 40.2% (202/503) were aged 35-54 years (34% of Facebook users in Canada), 14.5% (73/503) were aged 55-64 years (12% of Facebook users in Canada), and 7.2% (36/503) were aged ≥65 years (10% of Facebook users in Canada) [28]. Gender imbalance was present across all recruitment methods but especially so among social media recruits: in Montreal, 77.1% (388/503) of the social media recruits were women compared with an average of 68% (788/1158) recruited through all other means of recruitment; in Saskatoon, 76.1% (67/88) were women compared with 73.7% (232/315); and in Vancouver, 85.4% (82/96) were women compared with 67.9% (216/318). Our gender imbalance is in the higher range of the 22 studies reporting a gender split in a 2016 systematic review, for which the median proportion of women was 61.1% [10]. We did not anticipate such a gender imbalance, although research has shown that women tend to join [29] web surveys and volunteer their time more than men [30], which may explain why more women completed the surveys.

INTERACT engaged with community organizations and institutions that had already established communication with citizens to promote the study. Low-income populations were best recruited through partner newsletters, consistent with previous research that supports working with community partners to reach priority populations [8,22]. Contacting citizens through such partners may improve the receptivity and trust of the participants [8]. This requires that the research team develop relationships with community partners who work directly with marginalized groups. Building relationships with both advocacy organizations as knowledge users and service delivery organizations as recruitment partners requires early and ongoing engagement from the research staff throughout the project.

When evaluating the extent of bias by sociodemographic factors in recruitment methods, one should consider the sociodemographic characteristics of each recruitment method. For example, because there is a higher share of female Facebook users, a nonbiased recruitment among Facebook users would result in more women participating. Similarly, mailing lists tend to have more up-to-date information on homeowners than tenants. This means that mail campaigns may be more effective in recruiting homeowners. Certain community organizations may have relationships with priority populations, facilitating recruitment. It is important to be aware of the sociodemographic population characteristics that these methods do reach before drawing conclusions about recruitment bias. Furthermore, although it is useful to assess bias for each specific method, using a variety of recruitment methods will tend to increase reach across sociodemographic groups. Table 6 presents a summary of the results and lessons learned from the INTERACT recruitment campaigns.

**Table 6:** Summary of strengths and weaknesses of each recruitment method, as seen in the INTERACT^a^ study (lessons learned from INTERACT results).

Recruitment method	Strengths	Weaknesses
Mailed letters	Highest and quickest completion rates Effective at recruiting older populations Higher share of older men than other methods	Most expensive cost-per-completer rate
Social media	Generally cost-effective for recruiting a large cohort Effective in recruiting women and younger participants	Had the lowest and slowest completion rates
News media	Low cost-per-completer rate Effective at recruiting older participants	Low effectiveness for recruiting participants without a university degree Little control from research team to garner attention from media
Partner communications	High completion rate Effective in reaching priority population participants Effective for recruiting participants without a university degree Least expensive cost-per-completer rate	Slow completion rate Important investments in time for building trust with partners
Snowball recruitment	Ease of implementation through automated email campaigns	Tends to reinforce trends within sample composition, because referred participants resemble their peers

^a^INTERACT: Interventions, Research, and Action in Cities Team.

### Recruitment Method and Time Efficiency

One of the contributions of this study is that it provides a novel method to predict the number of daily recruits in a population-based recruitment effort, testing finite distributed lag weights for each recruitment approach. These results can inform the timing of different recruitment campaigns, including indications of their expected reach through time. We provide a detailed methodology, R syntax, and sample data on GitHub to facilitate the reproduction of this approach in other contexts. A systematic review [31] of modeling techniques used to predict recruitment to randomized clinical trials revealed a variety of modeling approaches, including Poisson and negative binomial models or Bayesian, time series, and Markov chain models. Using Poisson and negative binomial models does not capture the immediate rise in recruitment after special campaigns (eg, the peaks of recruited participants after wide-reach news media coverage). Bayesian, time series, and Markov chain analyses are less simple to reproduce [31]. With distributed lag weights as proposed in our study, ordinary least square models can be used [32]. Our model performed well in predicting daily recruitment, and recruitment-specific lags provided useful indications about temporal reach.

### Limitations

The INTERACT study requires considerable time and effort from the participants. Beyond recruitment methods, the messaging used can affect diversity in recruitment. We used a variety of hooks and angles to capture the participants’ attention. The impact of these factors was not assessed in this study. Moreover, differences in protocol in each city, notably compensation and prizes, may explain some of the variation in questionnaire completion rates among the cities. Future research may want to explore the impact of different types of messaging and visuals, including levels of participation and the impact of incentives on completion rates. It is possible that participants could have heard about the study from several sources, suggesting over- and underestimations and possible correction effects among methods. However, the model performs well in terms of cumulative recruitment; lag effects per method provide useful indications of the temporal dimensions of different recruitment approaches. Lower recruitment rates among priority populations are due to barriers such as distrust of participants and lack of knowledge in research, cultural beliefs and language issues [9], fear of stigmatization among those who may have engaged in high-risk behavior [8], issues related to low (technology) literacy, limited knowledge on the benefits the research might provide [33], privacy concerns, competing interests among busy participants [34], and lack of trust in web-based recruitment strategies [35]. The research team addressed these barriers in part by dedicating efforts to presenting the goals of the research and recruiting participants in person, connecting with community organizations that could promote the study among their clients and members, and providing phone or in-person assistance to participants answering questionnaires. Recruitment methods are only part of the equation for making participation more appealing and safer for all. Consequently, research teams should decide on the protocol at the outset and budget accordingly. Building trust and addressing logistical hurdles with priority populations are key goals for our next waves and should be considered at the forefront of any population health research.

Another limitation of the recruitment model is the inability to determine the sociodemographic profiles of the (unknown) exposed populations. Although Facebook Analytics provides profile statistics on the people reached through advertisements, such as sex, age, and geography, equivalent data for other recruitment methods were not available. For example, the number of people who are exposed to news media, snowball campaigns, or partner newsletters is unknown. Our model did not control for the demographic characteristics of those who were exposed to our campaigns.

### Conclusions

Our study provides detailed documentation of recruitment efforts and the costs of population baseline samples across 3 Canadian cities. We also provide a novel lag-based modeling approach to evaluate the effectiveness of different recruitment strategies, illustrated using data from Montreal. Different recruitment methods had different costs, returns, and possible biases, suggesting that diversifying recruitment methods are useful to increase reach and sample diversity. Local contexts should not be ignored, as shown by the differences among the cities. Research teams should keep detailed logs of recruitment activities and ask participants to report how they were recruited to improve reporting of recruitment efficiency and costs. With increasing opportunities to collect large-scale citizen science data stemming from web-based platforms, smartphones, or wearables, setting up comprehensive recruitment strategies and better understanding how and why citizens choose to participate or not is important for the future of population-based research.

## Appendix

Multimedia Appendix 1 A hypothetical example to calculate the lagged intensity measure.

Multimedia Appendix 2 Number of recruited participants, rate, and time of completion by city and recruitment method.

Multimedia Appendix 3 Demographic characteristics by recruitment method and city.

## Abbreviations

BRT: Bus Rapid Transit
FSA: forward sortation area
INTERACT: Interventions, Research, and Action in Cities Team
VERITAS: Visualization, Evaluation, and Recording of Itineraries and Activity Spaces
