# Supplementary material for: Recruiting Participants for Population Health Intervention Research: Effectiveness and Costs of Recruitment Methods for a Cohort Study
Source: J Med Internet Res. 2021 Nov 12;23(11):e21142. doi: 10.2196/21142 (PMC8663714; doi:10.2196/21142)
Supplement: Multimedia Appendix 1 [file jmir_v23i11e21142_app1.docx]

**Multimedia Appendix 1**

**Table S1:** Hypothetical example of how 2 days and 4 days lagged intensity measure is calculated

| Days | Facebook Reach | Elapsed days from end of the lag period to: | | | 2 days lag | Elapsed days from end of the lag period to: | | | 4 days lag |
| --- | --- | --- | --- | --- | --- | --- | --- | --- | --- |
|  |  | X1 | X2 | X3 |  | X1 | X2 | X3 |  |
| 1 | X1 | 2 |  |  | X1*(2/3) | 4 |  |  | X1* (4/10) |
| 2 | 0 | 1 |  |  | X1*(1/3) | 3 |  |  | X1* (3/10) |
| 3 | 0 |  |  |  | 0 | 2 |  |  | X1* (2/10) |
| 4 | X2 |  | 2 |  | X2*(2/3) | 1 | 4 |  | X1* (1/10)  + X2 * (4/10) |
| 5 | 0 |  | 1 |  | X2*(1/3) |  | 3 |  | X2 * (3/10) |
| 6 | X3 |  |  | 2 | X3*(2/3) |  | 2 | 4 | X2 * (2/10)  + X3 * (4/10) |
| 7 | 0 |  |  | 1 | X3*(1/3) |  | 1 | 3 | X2 * (1/10) + X3 *  (3/10) |
| 8 | 0 |  |  |  | 0 |  |  | 2 | X3 *  (2/10) |
| 9 | 0 |  |  |  | 0 |  |  | 1 | X3 *  (1/10) |
