# Supplementary material for: Recruiting Participants for Population Health Intervention Research: Effectiveness and Costs of Recruitment Methods for a Cohort Study
Source: J Med Internet Res. 2021 Nov 12;23(11):e21142. doi: 10.2196/21142 (PMC8663714; doi:10.2196/21142)
Supplement: Multimedia Appendix 2 [file jmir_v23i11e21142_app2.docx]

**Multimedia Appendix 2**

**Table S2-1:** Number of recruited participants per city by recruitment method

|  | Montreal  (n=1158) | Saskatoon  (n=315) | Vancouver  (n=318) | Total  (n=1791) |
| --- | --- | --- | --- | --- |
| Age |  |  |  |  |
| Mean (SD) | 45.4 (15.3) | 30.5 (11.8) | 55.5 (15.3) | 45.6 (16.3) |
| Median [Min, Max] | 43.0 [18.0, 85.0] | 27.0 [18.0, 70.0] | 56.0 [18.0, 88.0] | 45.0 [18.0, 88.0] |
| Missing | 0 (0%) | 130 (41.3%) | 0 (0%) | 130 (7.3%) |
| Heard about the study: |  |  |  |  |
| Mailed letters | 148 (12.8%) | 0 (0%) | 134 (42.1%) | 282 (15.7%) |
| News media | 226 (19.5%) | 4 (1.3%) | 0 (0%) | 230 (12.8%) |
| Social media | 503 (43.4%) | 88 (27.9%) | 96 (30.2%) | 687 (38.4%) |
| Partner communications | 91 (7.9%) | 126 (40.0%) | 1 (0.3%) | 218 (12.2%) |
| Snowball recruitment | 81 (7.0%) | 18 (5.7%) | 22 (6.9%) | 121 (6.8%) |
| Other | 109 (9.4%) | 79 (25.1%) | 65 (20.4%) | 253 (14.1%) |

**Table S2-2:**  Time from completion of eligibility to completion of health questionnaire

|  | **Montrea**l | **Saskatoon** | **Vancouver** | | **Victoria** | **Total** | |
| --- | --- | --- | --- | --- | --- | --- | --- |
| Days |  |  |  |  | |  | |
| Mean | 13.9 | 9.52 | 8.91 | 10.7 | | | 12.0 |
| SD | 38.7 | 19.9 | 19.7 | 24.7 | | | 32.3 |
| [Min, Max] | [0.00, 263] | [0.00, 125] | [0.00, 166] | [0.00, 132] | | | [0.00, 263] |

**Table S2-3:** Completion numbers and rates by city and recruitment method

|  | **Completed eligibility questionnaire** | | | **Completed eligibility and health questionnaire** | | | **Completion rate of health questionnaire** | | |
| --- | --- | --- | --- | --- | --- | --- | --- | --- | --- |
|  | Montreal | Saskatoon | Vancouver | Montreal | Saskatoon | Vancouver | Montreal | Saskatoon | Vancouver |
|  | (n=1536) | (n=402) | (n=380) | (n=1158) | (n=315) | (n=318) | (n=1536) | (n=402) | (n=380) |
| **Reported recruitment method** |  |  |  |  |  |  |  |  |  |
| **Mailed Letters** | 171 | 0 | 148 | 148 | 0 | 134 | 86.5% | NA | 90.5% |
| **Social Media** | 701 | 121 | 122 | 503 | 88 | 96 | 71.8% | 72.7% | 78.7% |
| **News Media** | 280 | 4 | 0 | 226 | 4 | 0 | 80.7% | 100.0% | NA |
| **Partner Communications** | 104 | 159 | 1 | 91 | 126 | 1 | 87.5% | 79.2% | 100.0% |
| **Snowball Recruitment** | 118 | 19 | 30 | 81 | 18 | 22 | 68.6% | 94.7% | 73.3% |
| **Other** | 162 | 99 | 79 | 109 | 79 | 65 | 67.3% | 79.8% | 82.3% |
