# Supplementary material for: Recruiting Participants for Population Health Intervention Research: Effectiveness and Costs of Recruitment Methods for a Cohort Study
Source: J Med Internet Res. 2021 Nov 12;23(11):e21142. doi: 10.2196/21142 (PMC8663714; doi:10.2196/21142)
Supplement: Multimedia Appendix 3 [file jmir_v23i11e21142_app3.docx]

# Multimedia Appendix 3:

**Table S3-1:** Montreal demographic characteristics by recruitment method^[[1]](#footnote-1)^

|  | **Mailed letters (n=148)** | **Social media (n=503)** | **News media (n=226)** | **Partner communications (n=91)** | **Snowball recruitment (n=81)** | **Other (n=109)** | **Total (n=1158)** |
| --- | --- | --- | --- | --- | --- | --- | --- |
| Age |  |  |  |  |  |  |  |
| 18-34 | 10(6.8%) | 192(38.2%) | 44 (19.5%) | 25 (27.5%) | 43 (53.1%) | 34 (31.2%) | 348 (30.1%) |
| 35-54 | 58(39.2%) | 202 (40.2%) | 74 (32.7%) | 37 (40.7%) | 21 (25.9%) | 45 (41.3%) | 437 (37.7%) |
| 55-64 | 48 (32.4%) | 73 (14.5%) | 62 (27.4%) | 17 (18.7%) | 8 (9.9%) | 16 (14.7%) | 224 (19.3%) |
| 65-88 | 32 (21.6%) | 36 (7.2%) | 46 (20.4%) | 12 (13.2%) | 9 (11.1%) | 14 (12.8%) | 149 (12.9%) |
| Education |  |  |  |  |  |  |  |
| Less than University degree | 56 (37.8%) | 93 (18.5%) | 24 (10.6%) | 9 (9.9%) | 14 (17.3%) | 18 (16.5%) | 214 (18.5%) |
| University degree | 46 (31.1%) | 184 (36.6%) | 88 (38.9%) | 32 (35.2%) | 31 (38.3%) | 38 (34.9%) | 419 (36.2%) |
| Graduate degree | 46 (31.1%) | 226 (44.9%) | 113(50.0%) | 48 (52.7%) | 36 (44.4%) | 53 (48.6%) | 522 (45.1%) |
| Gender |  |  |  |  |  |  |  |
| Man | 60 (40.5%) | 111 (22.1%) | 82 (36.3%) | 36 (39.6%) | 33 (40.7%) | 38 (34.9%) | 360 (31.1%) |
| Woman | 87 (58.8%) | 388 (77.1%) | 142 (62.8%) | 54 (59.3%) | 47 (58.0%) | 70 (64.2%) | 788 (68.0%) |
| Other | 0 (0%) | 2 (0.4%) | 2 (0.9%) | 1 (1.1%) | 0 (0%) | 1 (0.9%) | 6 (0.5%) |
| Income |  |  |  |  |  |  |  |
| $0 to $49,999 | 35 (23.6%) | 148 (29.4%) | 35 (15.5%) | 17 (18.7%) | 25 (30.9%) | 33 (30.3%) | 293 (25.3%) |
| $50,000 to $99,999 | 45 (30.4%) | 162 (32.2%) | 88 (38.9%) | 25 (27.5%) | 30 (37.0%) | 30 (27.5%) | 380 (32.8%) |
| $100,000 to $149,999 | 26 (17.6%) | 88 (17.5%) | 49 (21.7%) | 23 (25.3%) | 10 (12.3%) | 21 (19.3%) | 217 (18.7%) |
| $150,000 to $199,999 | 16 (10.8%) | 44 (8.7%) | 19 (8.4%) | 6 (6.6%) | 7 (8.6%) | 10 (9.2%) | 102(8.8%) |
| $200,000 or more | 7 (4.7%) | 23 (4.6%) | 15 (6.6%) | 10 (11.0%) | 5 (6.2%) | 9 (8.3%) | 69 (6.0%) |
| **Ethnicity** |  |  |  |  |  |  |  |
| White / Caucasian | 133(89.9%) | 459 (91.3%) | 222 (98.2%) | 84 (92.3%) | 69 (85.2%) | 98 (89.9%) | 1065(92.0%) |
| Indigenous / Aboriginal | <5(0.9%) | <5(0.6%) | 0(0%) | 0 (0%) | 0 (0%) | <5 (0.9%) | 5(0.4%) |
| Visible minorities | 16 (10.8%) | 56(11.1%) | 11 (4.9%) | 6 (6.6%) | 16 (19.8%) | 12 (11.0%) | 117 (10.1%) |

**Table S3-2:** Saskatoon demographic characteristics by recruitment method^[[2]](#footnote-2)^

|  | **Social media (n=88)** | **News media (n=4)** | **Partner communications**  **(n=126)** | **Snowball recruitment (n=18)** | **Other (n=79)** | **Total (n=315)** |
| --- | --- | --- | --- | --- | --- | --- |
| **Age** |  |  |  |  |  |  |
| 18-34 | 22 (25.0%) | 0 (0%) | 83 (65.9%) | 6 (33.3%) | 15 (19.0%) | 126 (40.0%) |
| 35-54 | 26 (29.5%) | 0 (0%) | 10 (7.9%) | 5 (27.8%) | 10 (12.7%) | 51 (16.2%) |
| 55-64 | 5 (5.7%) | 0 (0%) | 0 (0%) | 0 (0%) | 0 (0%) | 5 (1.6%) |
| 65-88 | 2 (2.3%) | 0 (0%) | 0 (0%) | 1 (5.6%) | 0 (0%) | 3 (1.0%) |
| **Education** |  |  |  |  |  |  |
| Less than University degree | 33 (37.5%) | 2 (50.0%) | 53 (42.1%) | 4 (22.2%) | 43 (54.4%) | 135 (42.9%) |
| University degree | 34 (38.6%) | 2 (50.0%) | 47 (37.3%) | 7 (38.9%) | 23 (29.1%) | 113 (35.9%) |
| Graduate degree | 19 (21.6%) | 0 (0%) | 22 (17.5%) | 7 (38.9%) | 10 (12.7%) | 58 (18.4%) |
| **Gender** |  |  |  |  |  |  |
| Man | 19 (21.6%) | 2 (50.0%) | 31 (24.6%) | 4 (22.2%) | 24 (30.4%) | 80 (25.4%) |
| Woman | 67 (76.1%) | 2 (50.0%) | 94 (74.6%) | 14 (77.8%) | 55 (69.6%) | 232 (73.7%) |
| Other | 1 (1.1%) | 0 (0%) | 1 (0.8%) | 0 (0%) | 0 (0%) | 2 (0.6%) |
| **Income** |  |  |  |  |  |  |
| $0 to $49,999 | 35 (39.8%) | 1(25.0%) | 69 (54.8%) | 3 (16.7%) | 32(40.5%) | 140 (44.4%) |
| $50,000 to $99,999 | 21 (23.9%) | 3 (75.0%) | 13 (10.3%) | 8 (44.4%) | 16 (20.3%) | 61(19.4%) |
| $100,000 to $149,999 | 17 (19.3%) | 0 (0%) | 10 (7.9%) | 3 (16.7%) | 7 (8.9%) | 37 (11.7%) |
| $150,000 to $199,999 | 3 (3.4%) | 0 (0%) | 8 (6.3%) | 2 (11.1%) | 6 (7.6%) | 19 (6.0%) |
| $200,000 or more | 1 (1.1%) | 0 (0%) | 5 (4.0%) | 0 (0%) | 2 (2.5%) | 8 (2.5%) |
| **Ethnicity** |  |  |  |  |  |  |
| White / Caucasian | 76 (86.4%) | <5 (75.0%) | 72 (57.1%) | 12 (66.7%) | 49 (62.0%) | 212 (67.3%) |
| Indigenous / Aboriginal | 6 (6.8%) | 0 (0%) | 5 (0.4%) | <5 (5.6%) | 8 (10.1%) | 20 (6.3%) |
| Visible minorities | 5 (5.7%) | 0 (0%) | 49 (38.9%) | <5 (16.7%) | 21 (26.6%) | 78 (24.8%) |

**Table S3-3:** Vancouver demographic characteristics by recruitment method^[[3]](#footnote-3)^

|  | **Mailed letters (n=134)** | **Social media (n=96)** | **Snowball recruitment  (n=22)** | **Other (n=65)** | **Total (n=318)^[[4]](#footnote-4)^** |
| --- | --- | --- | --- | --- | --- |
| **Age** |  |  |  |  |  |
| 18-34 | 0 (0%) | 24 (25.0%) | 9 (40.9%) | 7 (10.8%) | 40 (12.6%) |
| 35-54 | 33 (24.6%) | 45 (46.9%) | 8 (36.4%) | 20 (30.8%) | 106 (33.3%) |
| 55-64 | 41 (30.6%) | 17 (17.7%) | 3 (13.6%) | 13 (20.0%) | 74 (23.3%) |
| 65-88 | 60 (44.8%) | 10 (10.4%) | 2 (9.1%) | 25 (38.5%) | 98 (30.8%) |
| **Education** |  |  |  |  |  |
| Less than University degree | 38 (28.4%) | 16 (16.7%) | 3 (13.6%) | 13 (20.0%) | 71 (22.3%) |
| University degree | 39 (29.1%) | 41 (42.7%) | 13 (59.1%) | 21 (32.3%) | 114 (35.8%) |
| Graduate degree | 54 (40.3%) | 37 (38.5%) | 6 (27.3%) | 31 (47.7%) | 128 (40.3%) |
| **Gender** |  |  |  |  |  |
| Man | 60 (44.8%) | 14 (14.6%) | 8 (36.4%) | 19 (29.2%) | 101 (31.8%) |
| Woman | 74 (55.2%) | 82 (85.4%) | 14 (63.6%) | 45 (69.2%) | 216 (67.9%) |
| Other | 0 (0%) | 0 (0%) | 0 (0%) | 1 (1.5%) | 1 (0.3%) |
| **Income** |  |  |  |  |  |
| $0 to $49,999 | 15 (11.2%) | 17 (17.7%) | 3 (13.6%) | 17 (26.2%) | 53 (16.7%) |
| $50,000 to $99,999 | 34 (25.4%) | 29 (30.2%) | 3 (13.6%) | 10 (15.4%) | 76 (23.9%) |
| $100,000 to $149,999 | 23 (17.2%) | 17 (17.7%) | 7 (31.8%) | 17 (26.2%) | 64 (20.1%) |
| $150,000 to $199,999 | 13 (9.7%) | 6 (6.2%) | 0 (0%) | 6 (9.2%) | 25 (7.9%) |
| $200,000 or more | 26 (19.4%) | 16 (16.7%) | 5 (22.7%) | 2 (3.1%) | 49 (15.4%) |
| **Ethnicity** |  |  |  |  |  |
| White / Caucasian | 117 (87.3%) | 75 (78.1%) | 18 (81.8%) | 52(80.0%) | 263 (82.7%) |
| Indigenous / Aboriginal | <5 (0.7%) | <5 (1.0%) | 0 (0%) | 2 (3.1%) | 4 (1.3 %) |
| Visible minorities | 15 (11.2%) | 20 (20.8%) | <5 (18.2%) | 9 (13.8%) | 48 (15.1%) |

1. Missing responses: Education (n=3, 0.3%); Gender (n=4, 0.3%); Income (n=97, 8.4%); Ethnicity (n=14, 1.2%) [↑](#footnote-ref-1)
2. Missing responses: Age (n=130, 41.3%), Education (n=9, 2.9%); Gender (n=1, 0.3%); Income (n=50, 15.9%); Ethnicity (n=5, 1.6%) [↑](#footnote-ref-2)
3. Missing responses: Education (n=5, 1.6%); Gender (n=1, 0.3%); Income (n=51, 16.0%); Ethnicity (n=3, 0.9%) [↑](#footnote-ref-3)
4. Partner communications and news media are not shown because n<5 [↑](#footnote-ref-4)
